# Design of an artificial transcriptional system for production of high levels of recombinant proteins in tobacco (*Nicotiana benthamiana*)

**DOI:** 10.3389/fpls.2023.1138089

**Published:** 2023-02-23

**Authors:** Areum Yun, Joohyun Kang, Juhun Lee, Shi-Jian Song, Inhwan Hwang

**Affiliations:** Department of Life Sciences, Pohang University of Science and Technology, Pohang, Republic of Korea

**Keywords:** strong promoter, strong terminator, artificial transcription factor, recombinant protein production, *Nicotiana benthamiana*

## Abstract

Plants have recently received much attention as a means of producing recombinant proteins because they are easy to grow at a low cost and at a large scale. Although many plant protein expression systems have been developed, there remains a need for improved systems that deliver high yields of recombinant proteins. Transcription of the recombinant gene is a key step in increasing the yield of recombinant proteins. However, revealed strong promoters, terminators, and transcription factors that have been identified do not necessarily lead to high level production of recombinant proteins. Thus, in this study, a robust expression system was designed to produce high levels of recombinant protein consisting of a novel hybrid promoter, FM′M-UD, coupled with an artificial terminator, 3PRt. FM′M-UD contained fragments from three viral promoters (the promoters of *Mirabilis* mosaic caulimovirus (MMV) full-length transcript, the MMV subgenomic transcript, and figwort mosaic virus subgenomic transcript) and two types of *cis*-acting elements (four GAL4 binding sites and two zinc finger binding sites). The artificial terminator, 3PRt, consisted of the PINII and 35S terminators plus RB7, a matrix attachment region. The FM′M-UD promoter increased protein levels of reporters GFP, RBD : SD1 (part of S protein from SARS-CoV-2), and human interleukin-6 (hIL6) by 4–6-fold, 2-fold, and 6-fold, respectively, relative to those of the same reporters driven by the CaMV 35S promoter. Furthermore, when the FM′M-UD/3PRt expression cassette was expressed together with GAL4/TAC3d2, an artificial transcription factor that bound the GAL4 binding sites in FM′M-UD, levels of hIL6 increased by 10.7-fold, relative to those obtained from the CaMV 35S promoter plus the RD29B terminator. Thus, this novel expression system led to the production of a large amount of recombinant protein in plants.

## 1 Introduction

Plant molecular pharming is a promising way to produce valuable recombinant proteins such as protein drugs, vaccines, growth factors, hormones, and therapeutic antibodies in a cost-effective manner. Here, one key question is how to obtain a large quantity of the recombinant proteins. This question has been addressed at many different levels that include gene expression improvement, selection of host species with a high cell division rate, easy of recombinant protein purification, etc. Indeed, a great deal of efforts has been devoted to screen strong promoters in various plant species (Makhzoum et al., 2014; Kummari et al., 2020). The promoter from the Arabidopsis serine carboxypeptidase-like gene AtSCPL30 has been isolated as a potential tool for crop transgenic breeding (Jiang et al., 2018). A soybean (Glycine max) polyubiquitin promoter gave strong constitutive expression in transgenic soybean (Hernandez-Garcia et al., 2009). Cestrum yellow leaf curling virus (CmYLCV) promoter was identified as a strong constitutive promoter for heterologous gene expression in a wide variety of crops (Stavolone et al., 2003). However, the strength of the promoter has been shown to be dependent on plant species (Bhullar et al., 2009). Thus, proper selection of a promoter is important to achieve high level of proteins in a given plant species. Another important tool in the high-level gene expression is codon optimization, which also depends on the plant species. Various steps in post-translation also affect the accumulation of recombinant proteins. For example, efficient targeting using highly efficient leader sequence and the ER retention motif have been shown to be critical for high level accumulation in the ER. However, despite of these efforts, there should still be rooms to improve the production level of the recombinant proteins.

Transcription of a gene is usually regulated by a promoter located prior to the coding region in the 5′ upstream sequence. The promoter contains DNA sequences recognized by RNA polymerases and transcription factors, which together form part of the transcription machinery. Many different sequence motifs, also known as *cis*-acting elements, are often present in the promoter and required for correct transcription (Kadonaga, 2004; Li and Davidson, 2009). As a single promoter often contains many different types of *cis*-acting elements, and the same *cis*-acting elements are found in the promoters of many different genes, *cis*-acting elements often remain functional when they are transferred between promoters (Kadonaga, 2004) *Cis*-acting elements are recognized by *trans*-acting factors, such as transcription factors and other parts of the transcription machinery (Zhang et al., 2013). In general, transcription factors contain a DNA binding domain (DBD), which binds to specific *cis*-acting elements, and transcription activation domains (AD) (Brent and Ptashne, 1985). These domains in general act independently. The specificity of DBDs is conferred by the nucleotide sequences of *cis*-acting elements. ADs are responsible for interaction with transcription machinery, resulting in transcription (Boija et al., 2018).

As the promoter contains many different types of *cis*-acting elements, its “strength” is critically dependent on the particular combination present. The promoter may be divided into different functional regions: the core promoter, proximal promoter, and distal promoter. The core promoter is the nearest region to the start codon and contains the RNA polymerase (RNA pol) binding site, the transcription start site (TSS), and the TATA box (Burley, 1996) The binding of RNA pol to its binding site leads to transcription initiation (Vuthoori et al., 2001). The TATA box is bound by general transcription factors that boost the initiation of transcription (Burley, 1996). The proximal promoter is located upstream from the core promoter. It contains various primary regulatory elements bound by regulatory transcription factors (Maston et al., 2006). The distal promoter lies further upstream from the proximal promoter; it also contains many *cis*-acting elements bound by *trans*-acting factors (Bulger and Groudine, 2011). In general, the proximal promoter contains regulatory elements that function either as enhancers, which increase transcription levels *via* binding of activators, or as silencers that suppress transcription *via* binding of repressors (Levine and Tjian, 2003). All these various *cis*-acting elements contribute to promoter function, and their combined effects together determine the transcriptional strength of the promoter.

Much effort has been applied to identifying or designing strong promoters for biotechnology applications in plants (Xie et al., 2003; Xiao et al., 2005; Zhu et al., 2020); for example, a strong promoter was used to drive heterologous (foreign) genes in transgenic plants to obtain high levels of expression (Amack and Antunes, 2020). Viral genes are the source of many strong promoters. The cauliflower mosaic virus (CaMV) 35S promoter is a robust promoter that has been widely used in plants to express foreign genes at high levels (Amack and Antunes, 2020). The *cis*-acting elements of the CaMV 35S promoter are well characterized at the molecular level (Fang et al., 1989). Duplication of a region containing key *cis*-acting elements, named the double enhancer CaMV 35S promoter, leads to a further increase in promoter activity (Kay et al., 1987). The C1 promoter of cotton leaf curl Multan virus (CLCuMV) is another strong promoter used in transgenic plants. Use of the CLCuMV C1 promoter produces expression levels in transgenic tobacco leaves that are 3–5-fold higher than those produced by the CaMV 35S promoter (Xie et al., 2003). The full-length transcript promoter of dahlia mosaic virus (DaMV) is a constitutive promoter that is four times higher than the CaMV 35S promoter (Sahoo et al., 2014), and, in transgenic alfalfa plants, the cassava vein mosaic virus (CsVMV) promoter produces 24-fold higher expression level than the CaMV 35S promoter (Samac et al., 2004).

A strong promoter may also be obtained by constructing an artificial promoter. Specific *cis*-acting elements may be inserted into a heterologous promoter to induce additional binding of a cognate *trans*-acting factor, thereby strengthening promoter activity. Zinc finger binding sites or upstream activating sequences (UASs) from yeast genes can be inserted into promoters to increase their activity (Jamieson et al., 1994; VallsDe Lorenzo, 2003; Liang et al., 2006). In such cases, it is also necessary to introduce cognate transcriptional activators, such as GAL4/VP16, an artificial transcription activator that consists of the GAL4 DBD, which specifically recognizes UAS, and the herpes simplex virus transcriptional activating domain VP16 (Sadowski et al., 1988; Asakawa and Kawakami, 2008). The GAL4/UAS system has been widely used in various organisms, including both animals and plants (Halpern et al., 2008; Zhu et al., 2020).

In this study, we generated a strong promoter by combining three different approaches. We initially determined whether recombining fragments from certain viral promoters could produce a stronger promoter. Next, we examined whether insertion of an artificial sequence element, such as UAS or zinc finger binding sites, coupled with coexpression of cognate transcription factors further enhanced promoter strength. Finally, we asked whether particular terminators increased expression levels. The combination of three fragments from the promoters of the Mirabilis mosaic caulimovirus (MMV) full-length transcript (FLt), the MMV subgenomic transcript (Sgt), and figwort mosaic virus (FMV) Sgt produced a strong hybrid promoter, FM′M. A composite terminator sequence, PRt, consisting of the 35S terminator, the protease inhibitor II (PINII) terminator, and RB7, a matrix attachment sequence, increased the expression of genes driven by the FM′M promoter. Finally, inserting UAS×4 into the distal region of the FM′M promoter further enhanced expression levels when the promoter was coexpressed with an artificial transcription factor, GAL4:TAC3d2.

## 2 Materials and methods

### 2.1 Construction of plant expression vectors

The DNA fragments *MMV FLt* (-193 to +63 bp), *MMV Sgt* (-306 to -125 bp), and *FMV Sgt* (-270 to -63 bp) were chemically synthesized (Gene Universal, Inc., Newark, USA) (**Supplemental Table 1**). The FM′M promoter was first synthesized as a single DNA fragment. Subsequently, each domain was amplified by PCR using the specific primers MMFg-F, MMSg-F, FsMf-F, FsMf-R, M′FM-R, M′FM-F, and FM′M-R (**Supplemental Table 2**). To insert the UAS×4 motif and/or the single or double zinc finger binding motifs into the FM′M promoter, the primers FM′M-U-F and FM′M-R or FM′M-US-F and FM′M-US-R or FM′M-UD-F and FM′M-UD-R were used for overlapping PCR (**Supplemental Table 2**). All promoter fragments were digested with *PstI* and *XbaI*, and ligated into pCAMBIA1300, digested with the same restriction endonucleases. The *BiP : GFP:HDEL* recombinant construct (Islam et al., 2020) was ligated to each of the hybrid promoters following digestion with the restriction endonucleases *XbaI* and *XhoI.* The reporter gene encoding BiP : RBD:SD1:6×His : HDEL (Bangaru et al., 2020) was digested with the restriction endonucleases *XbaI* and *XhoI*, and ligated to the FM′M-UD or CaMV 35S promoters that had previously been digested with the same restriction endonucleases. The recombinant construct, *BiP : MP:CBM3:bdSUMO:hIL6:HDEL* (Islam et al., 2019), was ligated to the FM′M-UD or CaMV 35S promoters following digestion with *XbaI* and *XhoI* restriction endonucleases. All these constructs contained the RD29B terminator from *Arabidopsis thaliana RD29B* (D.13044.1) or the recombinant 3PR terminator (**Supplemental Table 1**). The 3PR terminator sequence was chemically synthesized (Gene Universal, Inc., Newark, United States). These terminators were ligated into the constructs after digestion with restriction endonucleases *XhoI* and *EcoRI.* The DNA fragments encoding the transcription factors GAL4:VP16, GAL4:TAC3d2, and ZinC7:TAC3d2 were chemically synthesized (Gene Universal, Inc., Newark, United States), and contained *XbaI* and *XhoI* restriction endonuclease sites. The MacT promoter and RD29B terminator were used for the expression of these transcription factors. All the primer sequences used in this study are listed in **Supplemental Table 2**.

### 2.2 Plant growth condition and *Agrobacterium*-mediated infiltration in *N. benthamiana*

*N. benthamiana* seeds were sown on soil and grown under the 16 h/8 h light/dark cycle at 25°C. 2 weeks-old seedlings were transferred to bigger pots and further grown under the 16 h/8 h light/dark cycle at 25°C for 3 weeks.

For *Agrobacterium*-mediated infiltration, expression constructs were introduced into *Agrobacterium tumefaciens* strain GV3101 by electroporation. Transformed *Agrobacteria* were grown in LB medium (Pronadias, Co:1551) at 28°C for 36 to 48 h, and cells were harvested by centrifugation at 2691 x g for 10 min and resuspended in infiltration buffer (10 mM MES, 10 mM MgSO_4_, 200 µM Acetosyringone, pH 5.6). Final cell density was adjusted to 0.5 of OD_600_. *Agrobacterium* suspension was incubated under the dark condition at room temperature without shaking for 1 h and 20 min. Leaf tissues were harvested at 3- or 5-days postinfiltration (dpi).

### 2.3 *Cis*-acting sequence element analysis

The database of PlantCARE(https://bioinformatics.psb.ugent.be/webtools/pmantcare/html) was used to analyze the 770 bp of the FM’M-UD promoter for potential *cis*-acting elements.

### 2.4 RNA extraction and quantitative real-time PCR

Total RNA was isolated from 100 mg of plant leaf tissue using the Qiagen RNeasy mini kit according to the manufacture’s protocol. Total RNA was treated with TURBO DNase (Invitrogen, MA, USA). 2 µg of total RNA were used for cDNA synthesis using the high-capacity cDNA reverse transcription kit (Applied Biosystems, MA, USA). qRT-PCR was carried out using 200 ng of cDNA (total reaction volume was 20 µg), hIL6-specific primers (**Supplemental Table 2**) and PowerUP SYBR Green Master Mix (Applied Biosystems, MA, USA). PCR was performed at the condition of 15 s denaturation at 95°C, 20 s annealing at 60°C and 30 s elongation at 70°C in a total of 40 cycles. *Hygromycin* resistant gene was amplified by primers, HygR-F and HygR-R, was used as an internal control for qRT-PCR (**Supplemental Table 2**).

### 2.5 Preparation of total soluble protein extracts, SDS-PAGE and Western blot analysis

Fresh leaves were ground under liquid nitrogen, and then mixed immediately with extraction buffer (50 mM Tris-HCl, pH 7.5, 150 mM NaCl, 1 mM EDTA, 1% [v/v] Triton X-100, and 1% protease inhibitor cocktail (Roche, Basel, Switzerland). Total soluble proteins (TSP) were obtained by centrifugation at 8000 x g for 10 min. Protein concentrations were measured by Bradford protein assay (Bio-Rad protein assay dye reagent, Bio-Rad, CA, USA).

Total soluble proteins were separated by 10% SDS/PAGE and analyzed by western blotting. We used different amounts of total soluble proteins depending on the target proteins to avoid saturation of signals on western blot images. For GFP, we used 500 ng of total soluble proteins whereas we used 10 μg of total soluble proteins for RBD : SD1 (part of S protein from SARS-CoV-2) and human interleukin-6 (hIL6). After SDS/PAGE, gels were stained using a staining solution containing 0.25% CBB R-250 (AMRESCO, USA, Pennsylvania), 45% methanol and 10% acetic acid.

For western blot analysis, membranes were blocked with 10% skim-milk (non-fat) in TTBS buffer (500 mM NaCl, 0.05% Tween-20, 20 mM Tris-HCl, pH 7.5) for 30 min, and incubated with mouse anti-hIL6 (Abcam, Cambridge, UK), rat anti-HA (Sigam, St.Louis, USA) or mouse anti-GFP (Clontech, Kusatsu, Japan) antibodies at a dilution of 1: 5000 in TTBS supplemented with 5% skim-milk at 9°C overnight. The membranes were washed twice with TTBS buffer. Anti-mouse IgG or anti-rat IgG as secondary antibody were incubated at 9°C for 2 h. Western bands were developed by using ECL solution (Neutronex, Goryeong, South Korea). For quantification, we measured the band intensity using image analysis software ImageJ (Rueden et al., 2017).

### 2.6 Purification of CBM3-hIL6 using CBM3-MCC affinity beads

To purify CBM3-hIL6 from extracts of total soluble protein (TSP), microcrystalline cellulose (MCC) beads (51 µm diameter; Sigma-Aldrich) were suspended in the same volume of distilled water. Next, 1 ml of TSP prepared from 100 mg of leaf tissue was mixed with 100 µl of MCC beads at 9°C for 1.5 h. The supernatant containing unbound proteins (UB) and pellet containing MCC beads were collected separately after centrifugation at 2000 × g for 1 min. The MCC beads were incubated with 1 ml extraction buffer by gentle shaking at 9°C for 10 min. The mixture was centrifuged for a second time, and, again, the MCC beads and supernatant were separately collected. The supernatant was saved as wash-off fraction (W). This process was repeated three times. Proteins bound to MCC beads were released in 6 × sample buffer (500 mM Tris-HCl, pH 6.8, 10% SDS, 0.5% bromophenol blue, 30% glycerol [v/v], and 100 mM DTT) by boiling for 10 min and then analyzed using SDS/PAGE followed by CBB staining or western blot analysis. 1% of TSP, UB, W1, and W2, and 10% of bound proteins, were loaded for western blot analysis and CBB gel staining.

## 3 Results

### 3.1 Design of novel hybrid promoters and their expression in *Nicotiana benthamiana*

The aim of this study was to develop a strong promoter capable of driving high levels of expression of foreign genes in tobacco (*Nicotiana benthamiana*) for the purpose of recombinant protein production. Many strong promoters have been identified from various plant viruses. However, these promoters may not be strong enough for the production of recombinant proteins for commercial purpose. For example, a previous study showed that the *Cotton Leaf Curl Burewala Virus* (CLCuBuV) C1 gene promoter exhibits 2-3 fold higher GUS activity than CaMV 35S promoter (Ashraf et al., 2014). However, when the CLCuBuV C1 promoter was used to express *BMCS:hIL6*, the expression level of BMCS:hIL6 was lower than that from CaMV 35S promoter (**Supplemental Figure 1**).

**Figure 1:**
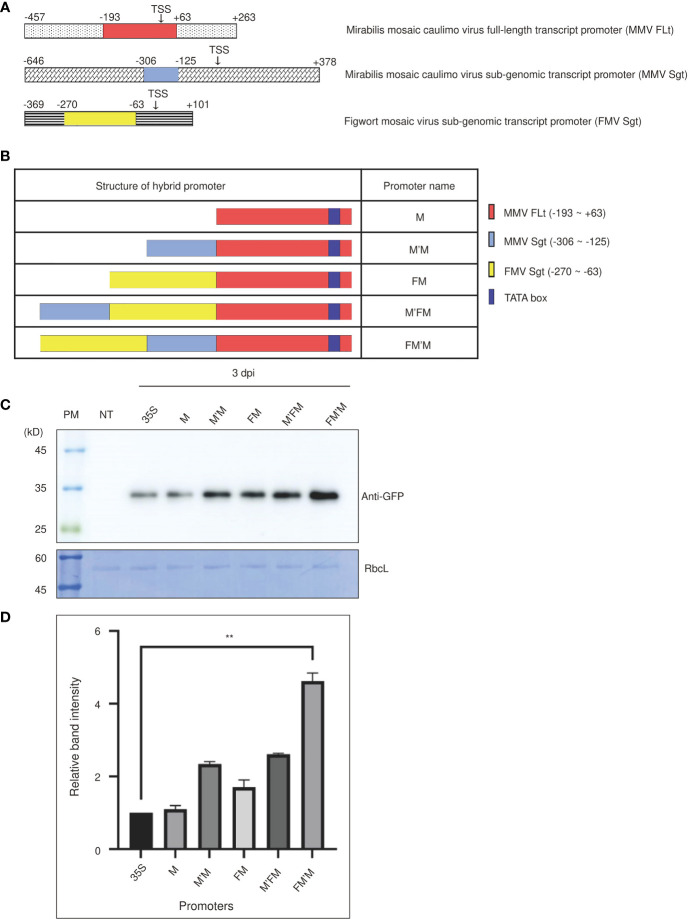
An artificial FM′M promoter generated by fusing fragments from three viral promoters induces strong expression of reporter genes in tobacco (*Nicotiana benthamiana*). **(A)** The promoter regions used to generate the FM′M promoter. The different regions making up the FM′M promoter are indicated in the full-length promoters of *MMV FLt* (M), *MMV Sgt* (M′), and *FMV Sgt* (F). Transcription start sites (TTS) are indicated. The numbers are the positions of nucleotide in the promoter; + and – indicate the downstream and upstream regions relative to the TTS, respectively. **(B)** Schematic presentation of hybrid promoters. The dark blue box indicates the TATA box. **(C, D)** Expression levels of the GFP reporter. The reporter constructs consisting of the indicated promoters and the reporter gene *GFP* were introduced into tobacco (*N. benthamiana*) leaf cells *via Agrobacterium*-mediated infiltration. Leaf tissues were harvested at 3 dpi. **(C)** Total soluble proteins from leaf tissues at 3 dpi were analyzed by western blotting using anti-GFP antibody. An identical membrane was stained with CBB; RbcL was used as a loading control. PM: protein size standard; NT: wild-type plant without infiltration; 35S: CaMV 35S promoter. **(D)** To quantify the GFP levels at 3 dpi, the band intensities were measured using the LAS-3000 imaging system. GFP levels from hybrid promoters are shown as values relative to that produced by the CaMV 35S promoter. Data shown are means ± SE (n = 3); asterisks indicate statistically significant differences determined by one-way ANOVA followed by Dunnett’s multiple comparison test; **: *P* < 0.01.

We aimed to construct a hybrid promoter that was stronger than any of its parental promoters by combining sequence domains containing *cis*-acting elements from different promoters. To do this, we selected three strong viral promoters as sources of building blocks to construct the hybrid promoter; these were the *Mirabilis* mosaic caulimovirus (MMV) full-length transcript (FLt) promoter, the MMV subgenomic transcript (Sgt) promoter, and the FMV subgenomic transcript (Sgt) promoter (Dey and Maiti, 1999; Bhattacharyya et al., 2002; Dey and Maiti, 2003). Although *cis*-acting elements are usually located in the upstream regions, certain promoters also contain *cis*-acting elements downstream of the transcription initiation site; for instance, the region of the MMV FLt promoter from the TSS to +63 base-pairs (bp) contributes to high levels of FLt expression (Dey and Maiti, 1999). In addition, there is a GATG repeat sequence, a core sequence for the enhancer element as-2 (Lam and Chua, 1989), at -189 to -182 bp relative to the TSS. We therefore used the -193 to +63 bp fragment of the MMV FLt promoter as a building block for the core promoter region of our new hybrid promoter. The MMV Sgt promoter sequence from -306 to -125 bp was used as the second building block. This upstream promoter fragment contains crucial *cis*-acting elements; its selection was informed by a previous set of promoter deletion experiments that showed this fragment contains important sequence elements determining promoter strength (Dey and Maiti, 2003). The final block used to build our novel promoter was the -63 to -270 bp fragment of the FMV Sgt promoter. Important sequence elements are present in this region of the FMV Sgt promoter (Bhattacharyya et al., 2002) (**Figure 1A**).

We generated various hybrid promoters using these three building blocks (**Figure 1B**). A DNA fragment MMV FLt (-193 to +63 bp), referred to as M, was used as the core region of the hybrid promoter. This fragment contained a TATA box, which is the core promoter region for initiation of transcription, and certain downstream elements (Dey and Maiti, 1999). The M fragment was fused first with either the MMV Sgt (-306 to -125 bp; referred to as M′) fragment to produce the M′M promoter or with the FMV Sgt (-270 to -63 bp; referred to as F) fragment to produce the FM promoter. Finally, we constructed promoters that contained all three building blocks by fusing F to M′M to produce FM′M, and M′ to FM to give M′FM. The cauliflower mosaic virus 35S promoter, a well-known robust promoter, was used as a control (Amack and Antunes, 2020). All the hybrid promoters were used to drive expression of a chimeric construct *BiP : GFP:HDEL*, where *BiP* and *HDEL* were the leader sequence from Arabidopsis *BiP1* for the ER targeting and ER retention motif, respectively (Islam et al., 2019).

We examined the strength of these promoters in tobacco. Recombinant *GFP* constructs were introduced into tobacco leaf tissues *via Agrobacterium*-mediated infiltration. A suspension culture of *Agrobacterium tumefaciens* harboring *p38*, a gene silencing suppressor from the turnip crinkle virus (Qu et al., 2003), was mixed at a 1:1 ratio with an *A. tumefaciens* suspension culture harboring *BiP : GFP:HDEL*. Leaves were harvested at 3 and 5 days postinfiltration (dpi), and total protein extracts were prepared for western blot analysis using anti-green fluorescent protein (GFP) antibody. The 53 kD Rubisco large subunit (RbcL) was used as a loading control (**Figure 1C**). GFP was detected at a position of approximately 35 kD. The five promoters increased levels of *BiP : GFP:HDEL* expression in the order M, M′M, FM and M′FM and FM′M. The M promoter produced similar levels of expression to the CaMV 35S promoter, but expression from the other hybrid promoters was stronger. To estimate the strength of the FM′M promoter, we quantified the intensity of the GFP expression driven by the FM′M and CaMV 35S promoters. The FM′M promoter showed a 4.5-fold higher protein level than the CaMV 35S promoter at 3 dpi (**Figure 1D**). The M′FM promoter, which contained the same fragments as FM′M but in a different order, was less active than the FM′M promoter, indicating that the position of *cis*-acting motifs was important for activating transcription (**Figure 1D**).

### 3.2 Insertion of *cis*-acting motifs into the hybrid FM′M promoter potentiates promoter activity

**Figure 2:**
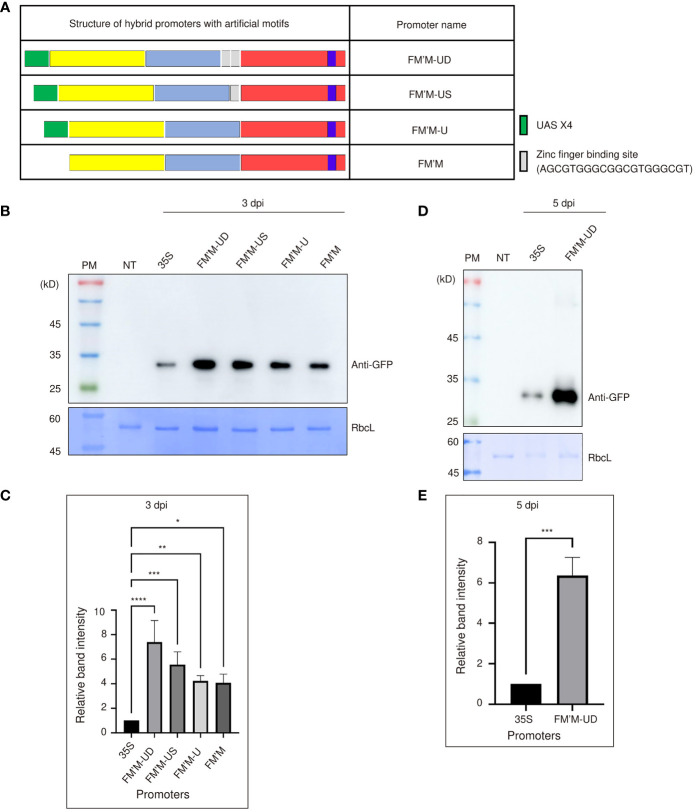
Insertion of UAS×4 and zinc finger binding sites enhances the strength of the FM′M promoter. **(A)** Schematic presentation of the hybrid promoter containing UAS×4 and zinc finger motifs. The sequence of the zinc finger motif is shown. **(B-E)** Expression level analysis of promoters. Reporter constructs consisting of the indicated promoters and *GFP* were transiently expressed in tobacco (*N. benthamiana*) leaves *via Agrobacterium*-mediated infiltration. **(B, D)** Total protein extracts (500 ng of total protein) were prepared from leaf tissues at 3 **(B)** or 5 **(D)** dpi and analyzed by western blotting using anti-GFP antibody. Identical membranes were stained with CBB; RbcL was used as a loading control. **(C, E)** To quantify the expression levels of GFP at 3 **(C)** and 5 **(E)** dpi, the GFP band intensities in the images shown in **(B, D)** were measured using the LAS-3000 imaging system; values shown are relative to that produced by the CaMV 35S promoter. Data shown are means ± SE (n = 3). Data were analyzed by one-way ANOVA followed by Dunnett’s multiple comparison test; asterisks indicate statistically significant differences; *: *P* < 0.05; **: *P* < 0.01; ***: *P* < 0.001; ****: P < 0.0001.

As sequence motifs can be inserted into a promoter to potentiate promoter activity (Dror et al., 2015), we next asked whether the hybrid promoter could be further strengthened by inserting additional *cis*-acting sequence elements. We tested the effects of inserting four copies of the UAS (UASx4) and/or the zinc finger binding motif, GCGTGGGCGGCGTGGGCG. UASx4 is bound by the yeast transcription factor GAL4; this UAS/GAL4 system has been widely used in various organisms, including plants (Aoyama and Chua, 1997; Akitake et al., 2011). The zinc finger binding motif has been used previously as an upstream *cis*-acting element for zinc finger-containing transcription factors to enhance transcription (Wu et al., 1995). We tested these motifs singly and in combination in the FM′M promoter. The FM′M-U promoter had only UAS×4 inserted at the 5′ end of the FM′M promoter, whereas the FM′M-US promoter contained UAS×4 plus a single copy of the zinc finger binding motif inserted between the FM′ and M fragments. Finally, the FM′M-UD promoter contained UAS×4 plus two copies of the zinc finger binding motif inserted between the FM’ and M fragments (**Figure 2A**). These promoters were each placed upstream of *BiP : GFP:HDEL*, and the resulting constructs were introduced to tobacco leaf tissues by *Agrobacterium*-mediated infiltration; a construct driven by the CaMV 35S promoter was used as a reference for expression. Total protein extracts were prepared from leaf tissues harvested at 3 dpi and analyzed by western blotting using anti-GFP antibody. RbcL stained with Coomassie brilliant blue (CBB) was used as a loading control (**Figure 2B**).

All these artificial promoters’ activity was more robust than the CaMV 35S promoter. Expression was increased by the addition of *cis-*acting motifs, even when transcription factors capable of binding these motifs were not coexpressed (**Figure 2B**). The FM′M-US and FM′M-UD promoters produced expression levels 4.5- and 6-fold higher expression, respectively, than the CaMV 35S promoter at 3 dpi (**Figure 2C**). The expression level of GFP driven by the FM′M-UD promoter was 6-fold higher than that of CaMV 35S promoter at 5 dpi; again, CBB-stained RbcL was used as a loading control (**Figures 2D, E**). Thus, insertion of these *cis*-acting elements enhanced promoter activity. This suggested that the original combination of sequence elements in the hybrid FM′M promoter may not have been optimal, but insertion of additional sequence elements between the core building blocks enhanced promoter activity, which in turn led to higher expression of the reporter gene.

**Figure 3:**
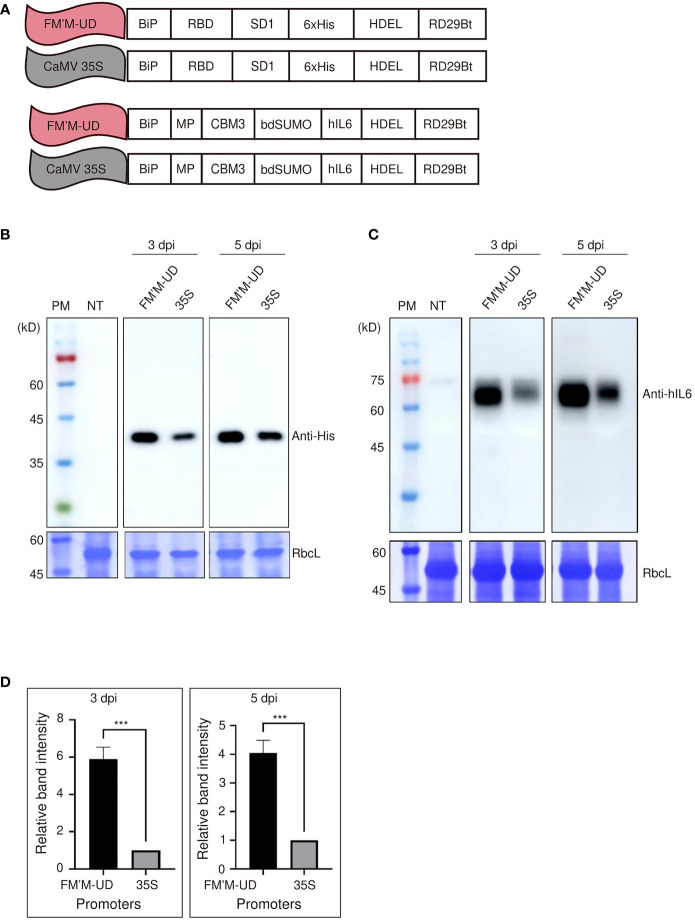
The FM′M-UD promoter induces strong expression of other reporters in *N. benthamiana*. **(A)** Schematic presentation of the reporter constructs containing *RBD : SD1* and *BMCS:hIL6*. **(B, C)** Expression analysis of the reporters at the protein level. Total soluble proteins were prepared from leaf tissues harvested at 3 and 5 dpi, and used in western blot analyses using anti-His **(B)** and anti-hIL6 **(C)** antibodies. As a loading control, identical membranes were stained with CBB to detect the level of RbcL. **(D)** Quantification of protein expression levels. To quantify the expression levels driven by the hybrid promoter and CaMV35S promoter, the band intensities of western blot images in **(C)** were measured using the LAS-3000 imaging system. The expression levels from the FM′M-UD promoter are relative to that produced by the CaMV 35S promoter. The data shown are means ± SE (n = 3). Asterisks shown in **(D)** indicate statistically significant differences determined by two-tailed Student’s *t*-tests; ***: *P* < 0.001.

To obtain further evidence for the enhanced strength of these promoters, we tested the activity of the FM′M-UD promoter using other reporter genes. Recent interest has focused on expressing recombinant S protein from SARS-CoV-2 (the virus responsible for COVID-19) in plants. The RBD : SD1 of the S protein plays a key role in the infection of human cells by SARS-CoV-2 *via* binding to ACE2 (Lan et al., 2020). Thus, *RBD : SD1* was designated a gene of interest (GOI). It was tagged with six histidine residues (6×His) and placed under the FM′M-UD promoter; as a control, *RBD : SD1:6×His* was also placed under the CaMV 35S promoter (**Figure 3A**). These constructs were introduced into tobacco leaf cells, and the levels of RBD-SD1:6×His were measured at 3 and 5 dpi by western blot analysis using anti-His antibody. The FM′M-UD promoter produced much higher levels of RBD : SD1:6×His expression than did the CaMV 35S promoter at both 3 and 5 dpi, confirming that FM’M-UD was a robust promoter. CBB-stained RbcL was used as a loading control (**Figure 3B**).

We further tested the activity of the FM’M-UD promoter using human interleukin 6 (hIL6) as a GOI. We showed previously that a recombinant human IL6 (hIL6) construct, *BiP : MP:CBM3:bdSUMO:hIL6:HDEL* (in short, *BMCS:hIL6*), is readily expressed in tobacco (Islam et al., 2019). We placed *BMCS:hIL6* under the control of either the FM′M-UD promoter to give *FM′M-UD::BiP : MP:CBM3:bdSUMO:hIL6:HDEL* (in short, *FM′M-UD::BMCS:hIL6*) or the CaMV 35S promoter to produce *CaMV35S::BiP : MP:CBM3:bdSUMO:hIL6:HDEL* (in short, *35S::BMCS:hIL6*) (**Figure 3A**). These constructs were introduced into tobacco leaf cells *via Agrobacterium*-mediated infiltration. Total protein extracts were prepared from leaf tissues at 3 and 5 dpi and analyzed by western blotting using anti-hIL6 antibody. The recombinant protein BMCS:hIL6 was detected at a position of approximately 65 kD. The expression levels of BMCS:hIL6 driven by the FM′M-UD promoter were 6- and 4-fold higher than that driven by the CaMV 35S promoter at 3 and 5 dpi, respectively; CBB-stained RbcL was used as a loading control (**Figures 3C, D**).

We performed an *in silico* analysis of *cis*-acting elements to determine how FM′M-UD might act as a powerful promoter inducing high rates of transcription. This analysis identified many *cis*-acting elements in the hybrid promoter FM′M. A WRE3 binding site, CAAT box, an as-1 element, a MYB recognition site, and an A/T-rich region were found in the FMV Sgt (-270 to -63 bp) and MMV Sgt (-306 to -125 bp) fragments (**Supplemental Figure 2**). The CAAT motif, which is present in the promoters of many higher eukaryotic genes, is a binding site for CBF, one of the core binding factors, and is essential for transcription (Bi et al., 1997; Laloum et al., 2013). The A/T-rich region, a recognition site for TFIID, is important for the accurate initiation of transcription, as well as to ensure a basal level of transcription (Sugihara et al., 2011).

### 3.3 An artificial transcription factor that recognizes UAS×4, but not zinc finger motifs, greatly enhances transcription from the promoter FM′M-UD

**Figure 4:**
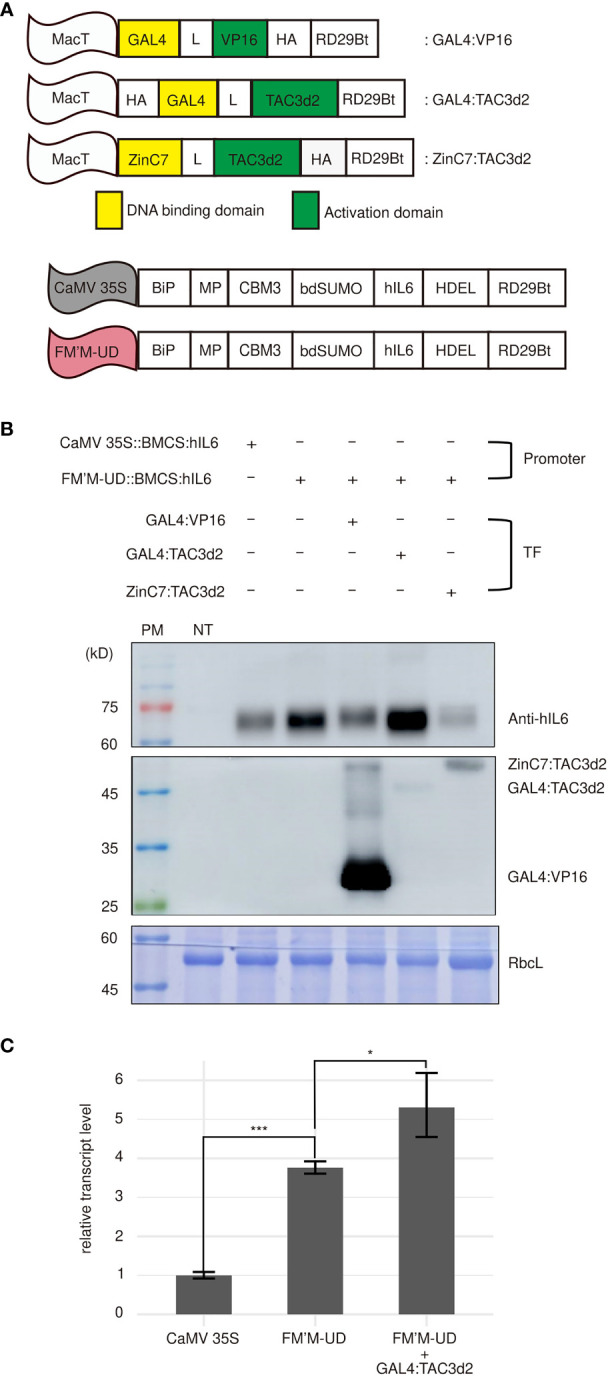
Artificial *trans*-activator-mediated stimulation of transcription from the FM′M-UD promoter depends on the type of activator domains. **(A)** Schematic presentation of artificial *trans*-activators used in this study. MacT is the promoter. DBDs are shown in yellow. HA: a small hemagglutinin tag used to detect the expression of artificial transcription factors; MP: a fragment containing multiple N-glycosylation sites from the human protein tyrosine phosphatase receptor type C (CD45); CBM3: cellulose-binding module 3; bdSUMO: SUMO domain of *Brachypodium*; hIL6: human interleukin 6; HDEL: an ER retention motif. **(B)** Expression levels of the reporter proteins and *trans*-activators. The indicated constructs were introduced into tobacco leaf cells by *Agrobacterium*-mediated infiltration. Total soluble proteins were prepared from leaf tissues harvested at 3 dpi and analyzed by western blotting using anti-hIL6 and anti-HA antibodies. **(C)** Comparison of the *hIL6* transcript levels produced by the CaMV 35S, FM’M-UD promoters and/or artificial transactivator GAL4:TAC3d2. A *hygromycine* resistant gene in the same vector was used as an internal control. The data shown are means ± SD (n = 3). Asterisks indicate statistically significant difference determined by two-tailed Student’s *t*-tests; *: *P* < 0.05; ***: *P* < 0.001.

Next, we asked whether coexpression of transcription factors that recognized the inserted *cis*-acting elements could further strengthen promoter activity. We generated several artificial transcription factors capable of recognizing the UAS×4 and zinc finger motifs. The chimeric transcription factor GAL4:VP16 binds the yeast UAS in plants (Jia et al., 2007). We added a small epitope, HA, to the C-terminus of GAL4:VP16 to produce the tagged protein GAL4:VP16:HA for detection by western blot analysis. The GAL4:VP16 *trans*-activator is less potent in inducing transcription when the UAS is located distantly from the TATA box (Zhu et al., 2020). In the FM′M-UD promoter used in this study, the UAS×4 motif was located at the distal end of the promoter. We therefore generated another artificial transcription factor, HA : GAL4:TAC3d2, using a duplicated transcription activation domain from TAC3, as described by Zhu et al. (2020). TAC3 is a transcription factor from Chinese fir (*Cunninghamia lanceolata*) (Zhu et al., 2020). When fused to GAL4, the TAC3d2 activation domain was found to be a stronger activator of transcription than VP16. In addition, we generated an artificial zinc finger transcription factor that bound the zinc finger binding site. The mutated murine transcription factor Zif268:C7:C7 binds specifically to the zinc finger motif GCGTGGGCGGCGTGGGCG with a very low dissociation constant (0.46 nM Kd) (Wu et al., 1995; Liu et al., 1997). We therefore fused Zif268:C7:C7 with TAC3d2 to produce ZinC7:TAC3d2, an artificial transcription factor containing a DBD that would recognize the zinc finger binding site in the FM′M-UD promoter (**Figure 4A**).

To examine the effect of these three artificial transcription factors, GAL4:TAC3d2, GAL4:VP16, and ZinC7:TAC3d2, on the expression of reporters under the control of the FM′M-UD promoter, *FM’M-UD::BMCS:hIL6* was coexpressed with each of the transcription factor constructs. Coexpression with *GAL4:TAC3d2* greatly enhanced expression of *BMCS:hIL6* from the FM′M-UD promoter (**Figure 4B**), indicating that GAL4:TAC3d2 was able to recognize the UAS×4 motif in the FM′M-UD promoter and stimulate transcription, even when UAS×4 was distally located from the TATA box. However, coexpression of *GAL4:VP16*, which also binds UAS×4, did not increase expression of *BMCS:hIL6* from the FM′M-UD promoter. This may be because the mode of activation of transcription differed between TAC3d2 and VP16, such that TAC3d2, but not VP16, could activate transcription from a remote site. Coexpression with *ZinC7:TAC3d2* resulted in lower expression of *BMCS:hIL6* from the FM′M-UD promoter than when the promoter construct was expressed alone, indicating that binding of ZinC7:TAC3d2 to the double zinc finger binding site functioned as a suppressor. The levels of transcription factors were detected using anti-HA antibody. GAL4:VP16 was expressed at a high level. The two other transcription factors, GAL4:TAC3d2 and ZinC7:TAC3d2, were also expressed (**Figure 4B**). CBB-stained RbcL was used as a loading control (**Figure 4B**).

We then examined whether high-level protein production by the FM’M-UD promoter with the artificial transactiviator GAL4:TAC3d2 is caused by an increase in the transcriptional level. Total RNA was extracted from leaf tissues (*N. benthamiana*) expressing *BMCS:hIL6* under the control of CaMV 35S promoter or FM’M-UD promoter and/or the GAL4/TAC3d2 transactivator and analyzed by quantitative RT-PCR (qRT-PCR). The *BMCS:hIL6* transcript level driven from the FM’M-UD promoter in the presence of coexpressed GAL4/TAC3d2 transactivator was 5.5 times higher than that from the CaMV 35S promoter. (**Figure 4C**). These results suggest that a combination of the AD and DBD in a chimeric transcription factor, as well as the location of the *cis*-acting elements, was important for transcriptional activation.

### 3.4 A composite terminator 3PR further enhances expression of reporters driven by the FM′M-UD promoter

**Figure 5:**
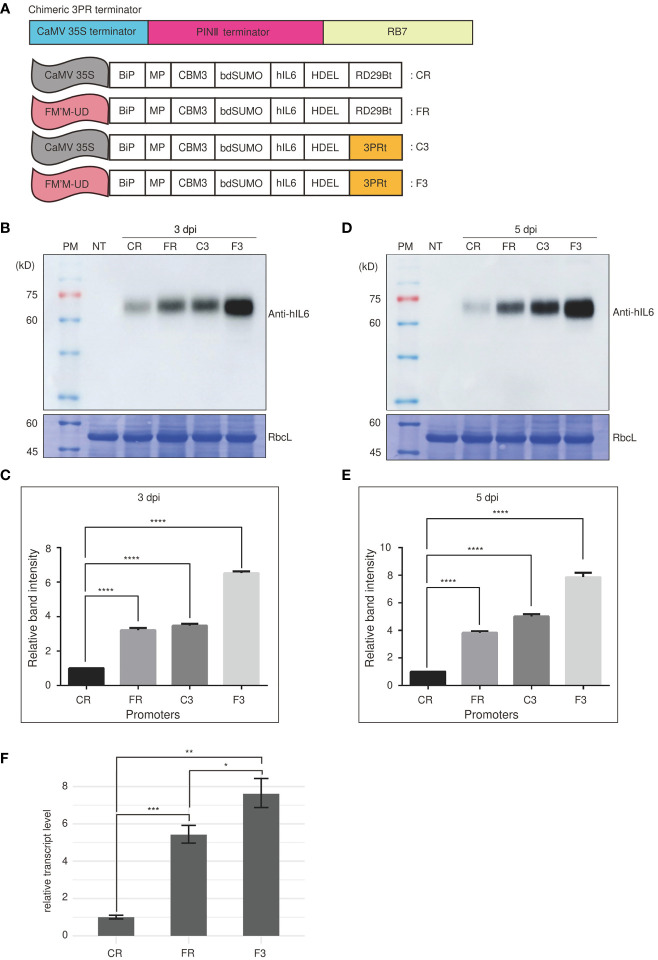
An artificial hybrid terminator, 3PRt, strongly enhances expression driven by the FM′M promoter. **(A)** Schematic presentation of the 3PR terminator and reporter constructs. **(A)** DNA fragments from the CaMV 35S terminator, the PINII terminator, and RB7 were fused to produce the 3PR terminator (3PRt). **(A)** The different expression constructs used in this study. CR: CaMV 35S promoter plus RD29Bt; FR: FM′M-UD promoter plus RD29Bt; C3: CaMV 35S promoter plus 3PRt; and F3: FM′M-UD promoter plus 3PR terminator. **(B-E)** Analysis of the expression level of reporter proteins. Leaf tissues of *N. benthamiana* were infiltrated with the indicated constructs. Total soluble proteins were prepared from leaf tissues harvested at 3 **(B, C)** and 5 dpi (**D, E**), and analyzed by western blotting using anti-hIL6 antibody. Identical membranes were stained with CBB to visualize RbcL, used as a loading control (**B, D**). To quantify the expression levels at 3 and 5 dpi, the band intensities of immunoblot images in **(B, D)** were measured using the LAS-3000 imaging system; values shown are relative to that produced by the CaMV 35S promoter. The data shown are means ± SE (n = 3); asterisks indicate statistically significant differences determined using one-way ANOVA followed by Dunnett’s multiple comparison test; ****: *P* < 0.0001. **(F)** Comparison of the *hIL6* transcript levels produced by the CR, FR and F3. A *hygromycine* resistant gene in the same vector was used as an internal control. The data shown are means ± SD (n = 3). Asterisks indicate statistically significant difference determined by two-tailed Student’s *t*-tests; *: *P* < 0.05; **: *P* < 0.01; ***: *P* < 0.001.

We next examined the effect of terminators on the expression of a reporter gene under the control of FM′M-UD. The transcription terminator plays a crucial role in controlling the expression of a gene (Carswell and Alwine, 1989; Ingelbrecht et al., 1989). A strong terminator may enhance gene expression by as much as 10-fold (Ingelbrecht et al., 1989; Nagaya et al., 2010). We generated an artificial terminator, the 3PR terminator (3PRt), using sequence fragments from the CaMV 35S terminator; a 3′ region of soybean protease inhibitor II (PINII) terminator; and a scaffold attachment region, RB7. We produced expression cassettes consisting of the FM′M-UD promoter plus either the 3PR terminator or the RD29B terminator (**Figure 5A**), and inserted a GOI, *BMCS:hIL6*, into these expression cassettes to produce the constructs *FM’M-UD::BMCS:hIL6::3PR* (in short, *F3*) and *FM’M-UD::BMCS:hIL6::RD29B* (in short, *FR*). These constructs were introduced into tobacco leaf cells using *Agrobacterium*-mediated infiltration. The expression constructs *CaMV35S*::*BMCS:hIL6::RD29B* (in short, *CR*) and *CaMV35S*::*BMCS:hIL6::3PR* (in short, *C3*) were included as controls (**Figure 5A**). To examine the effects on the expression levels of BMCS:hIL6, total protein extracts were prepared from leaf tissues harvested at 3 or 5 dpi and analyzed by western blotting using anti-hIL6 antibody. RbcL was used as loading control. Expression of BMCS:hIL6 was increased in the order CR, FR, C3, and F3 after both 3 and 5 dpi (**Figures 5B, D**). In particular, F3 greatly enhanced expression of BMCS:hIL6. When the bands were quantified, expression from F3 was 7- and 8-fold higher than expression from CR at 3 and 5 dpi, respectively (**Figures 5C, E**).

Next, we examined the effect of the 3PR terminator on the transcript level of *BMCS:hIL6* driven from the FM’M-UD promoter. We performed qRT-PCR analysis of the transcript levels. The three constructs, CR, FR and F3, were transiently expressed in *N.benthamiana* leaves *via Agrobacterium*-mediated infiltration. Total RNA from leaf tissues harvested at 3 dpi was used for qRT-PCR. The transcript level of *BMCS:hIL6* from the FM’M-UD promoter and the 3PR terminator was 7.5 fold to that from the CaMV 35S promoter and RD29B terminator (**Figures 5F**). These results indicated that the terminator was as important as the promoter in determining the level of expression.

### 3.5 Combining the FM′M-UD promoter, 3PR terminator, and GAL4/TAC3d2 transcription factor leads to a high yield of recombinant protein

**Figure 6:**
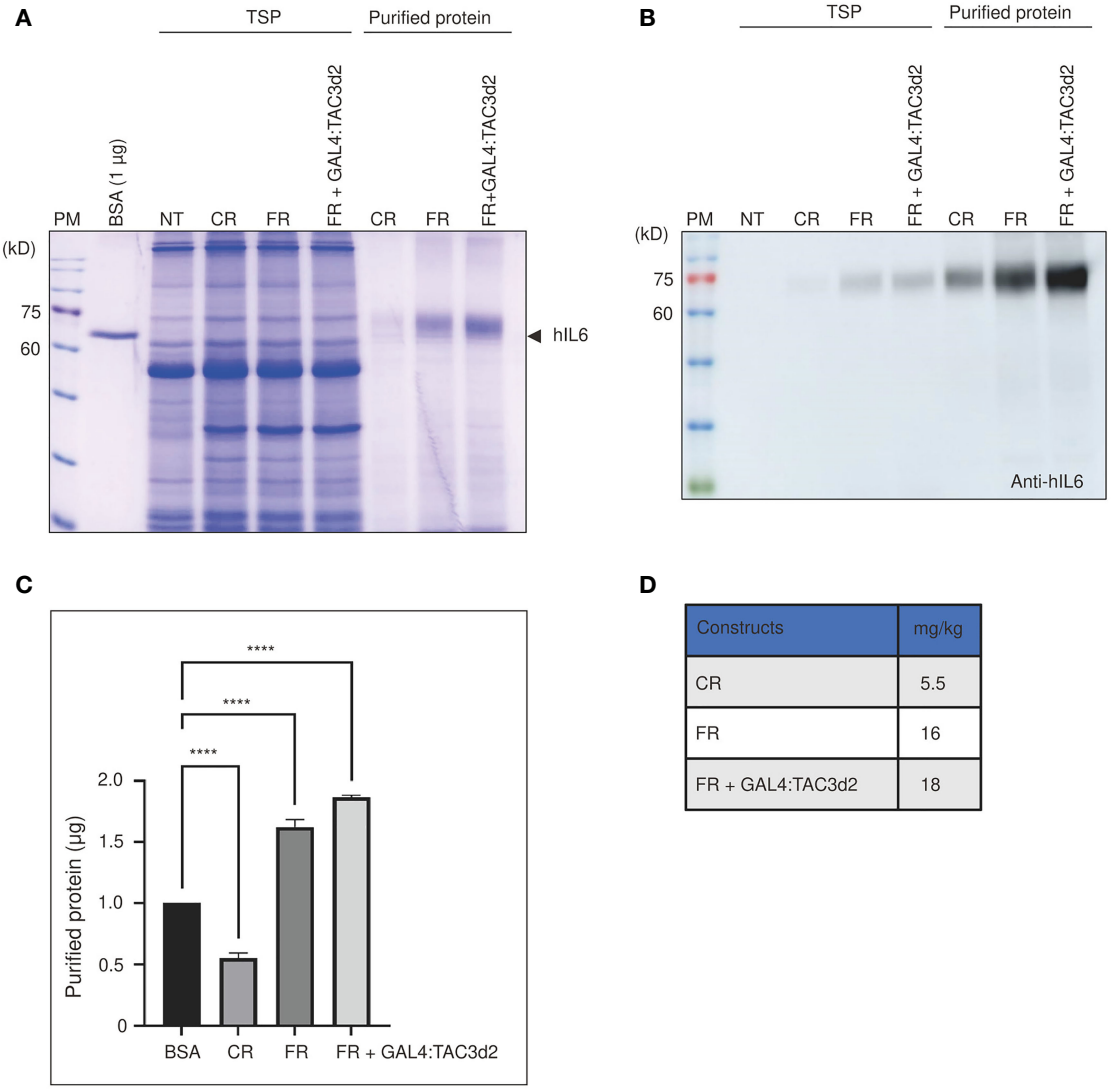
Purified proteins confirm the strong transcriptional activity from the hybrid FM’M-UD promoter with artificial GAL4/TAC3d2 transcription factor. **(A, B)** Analysis of the expression levels of purified proteins. Total soluble proteins (TSP) were extracted from 100 mg fresh leaf tissue harvested at 5 dpi and purified using MCC beads. BMCS:hIL6 bound to MCC beads was released by boiling and analyzed using SDS/PAGE followed by CBB staining **(A)** or western blot analysis using anti-hIL6 antibody **(B)**. BSA (1 µg) was used as a reference protein for quantification. NT: wild-type plant without infiltration. CR: CaMV 35S promoter plus RD29Bt; FR: FM′M-UD promoter plus RD29Bt; GAL4/TAC3d2: Transactivator consisting of GAL4 DNA binding domain plus TAC3d2 activation domain. **(C, D)** Quantification of purified proteins expressed from reporter constructs. The band intensities of purified recombinant proteins were measured using the LAS-3000 imaging system and converted to µg based on the intensity of 1 µg of BSA. The amount of protein produced from the indicated promoters is shown in mg/kg FW. The data shown are means ± SE (n = 3); asterisks indicate statistically significant differences measured using one-way ANOVA followed by Dunnett’s multiple comparison test; ****: *P* < 0.0001.

To assess the production yield of hIL6 from FM’M-UD promoter with coexpressed GAL4/TAC3d2, we purified target proteins using microcrystalline cellulose (MCC) beads as an affinity resin. Recombinant hIL6 protein contains the CBM3 domain that binds specifically to MCC beads (Islam et al., 2019). Total soluble protein extracts from tobacco leaves harvested at 5 dpi were incubated with MCC beads at a 10:1 (v/w) ratio. The MCC bead and supernatant fractions were collected separately following incubation. The supernatant contained proteins that did not bind to MCC beads (the unbound fraction; UB). The beads were washed three times with extraction buffer. Proteins bound to MCC beads (bound fraction) were eluted by boiling for 10 min in SDS/PAGE sample buffer. Both fractions were separated by SDS/PAGE. Gels were analyzed by staining with CBB (**Figure 6A**) and by western blotting using anti-hIL6 antibody (**Figure 6B**). Most of the host proteins were detected in the unbound fraction. A minor portion was also detected in wash 1 (W1) but not in W2, indicating that loosely bound host proteins are removed by two washing steps (**Supplemental Figure 3**). The amount of recombinant hIL6 increased according to which promoter construct was used, in the order CR, FR, and FR plus GAL4:TAC3d2 (**Figure 6C**). Protein quantifications based on the intensities of CBB-stained bands indicated yields of recombinant hIL6 proteins from CR, FR, and FR plus GAL4:TAC3d2 of 5.5, 16, and 18 mg/kg fresh weight, respectively (**Figure 6D**).

**Figure 7:**
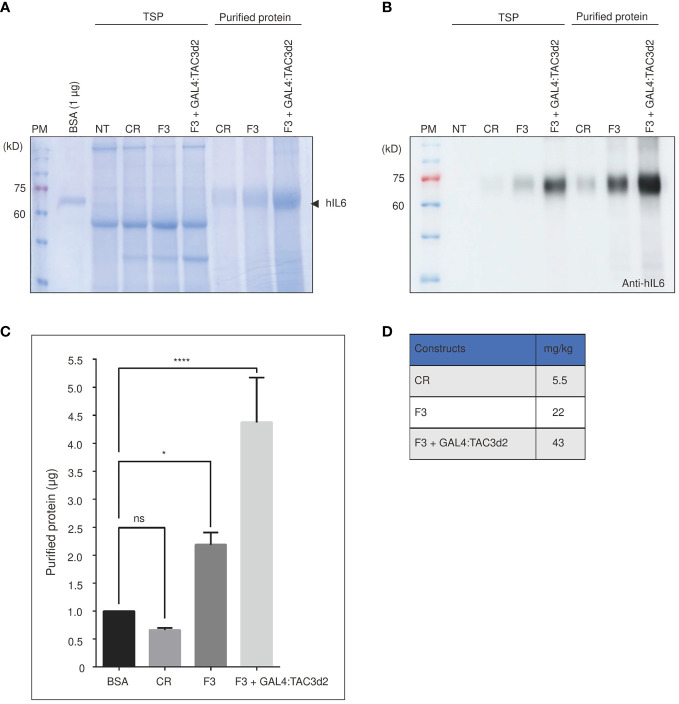
Protein purification confirms the composite terminator strongly enhances protein expression with FM’M-UD promoter and artificial transactivator GAL4/TAC3d2. **(A, B)** Analysis of purified report proteins. The reporter protein was purified using MCC beads from total soluble protein extracted from 100 mg leaf tissues and analyzed by SDS/PAGE followed by CBB staining **(A)** or western blotting using anti-hIL6 antibody **(B)**. NT: wild-type plant without infiltration. CR: CaMV 35S promoter plus RD29Bt; F3: FM′M-UD promoter plus 3PRt; GAL4/TAC3d2: Transactivator consisting of GAL4 DNA binding domain plus TAC3d2 activation domain. Arrow indicates purified BMCS:hIL6. **(C, D)** Quantification of purified proteins. The intensities of CBB-stained protein bands were measured using the LAS-3000 imaging system and converted to µg based on the intensity of 1 µg BSA. The yield was calculated based on the yield in **(C)** and is presented in mg/kg fresh weight (FW) **(D)**. Three independent experiments were carried out to quantify the production yield. The data shown are means ± SE (n = 3); asterisks indicate statistically significant differences determined using one-way ANOVA followed by Dunnett’s multiple comparison test; ns: no significant difference; *: *P* < 0.05; ****: *P* < 0.0001.

Next, we estimated the yield of proteins produced by the construct containing both the FM′M-UD promoter and the 3PR terminator (F3). Recombinant hIL6 protein was purified from total protein extracts from leaf tissues harvested at 5 dpi. Purified protein was analyzed by SDS/PAGE (**Figures 7A, B**), and the band intensity was measured to quantify the yield. F3 plus GAL4:TAC3d2 produced 43 mg/kg fresh weight of recombinant hIL6 protein. Thus, F3 plus GAL4:TAC3d2 generated a yield approximately 8-fold or 2-fold higher than CR or F3, respectively (**Figures 7C, D**). A minor portion was also detected in wash 1 (W1) but not in W2, indicating that loosely bound host proteins are removed by two washing steps (**Supplemental Figure 4**). Our expression cassette consisting of a hybrid promoter plus a composite terminator, when expressed in tobacco together with an artificial robust transcription factor GAL4:TAC3d2, produced high levels of recombinant proteins.

## 4 Discussion

We aimed to build a strong promoter capable of producing high levels of recombinant proteins in tobacco plants. Our strategy was to analyze three viral promoters to obtain a detailed understanding of their *cis*-acting elements and then recombine their most critical regions in different ways to generate a set of hybrid promoters. The promoter fragments we identified were FMV Sgt (-270 to -63 bp; F), MMV Sgt (-306 to -125 bp; M′), and MMV FLt (-193 to +63 bp; M) (Dey and Maiti, 1999; Bhattacharyya et al., 2002; Dey and Maiti, 2003). MMV FLt (-193 to +63 bp) contains a fairly long region downstream of the transcription initiation site (Dey and Maiti, 1999). Usually, the transcription initiation site occurs 20–30 base-pairs downstream of the TATA box; however, *cis*-acting elements downstream of the initiation site in the MMV FLt promoter also contribute to promoter activity (Dey and Maiti, 1999). We found that the strength of the hybrid promoters depended on the order in which these fragments were arranged. The FM′M promoter was stronger than M′FM, and both FM′M and M′FM were stronger than FM or M′M; this suggested that additional *cis*-acting elements in the upstream region were important for increasing promoter activity. *cis*-acting elements may be present in the distal promoter, an upstream region distant from the TATA box (Yanai et al., 1996). All the hybrid promoters, including M, were stronger than the CaMV 35S promoter, with the FM′M promoter being 4-fold stronger at the protein level. *In silico* analysis revealed that FM′M contained many known *cis*-acting elements, including a WRE3 binding site, a CAAT box, an as-1 element, a MYB binding site, and an A/T-rich region. The as-1 element is a key *cis*-acting element in the CaMV 35S promoter, whereas the CAAT box and AT-rich sequence are potent *cis*-acting elements for the induction of transcription (Bi et al., 1997; Sugihara et al., 2011; Laloum et al., 2013). An analysis of whether these *cis*-acting elements contributed to transcription in tobacco was, however, beyond the scope of this study.

In addition, we explored the effect of introducing additional *cis*-acting elements to a hybrid promoter. One of the best characterized *cis*-acting elements is the UAS bound by the yeast protein, GAL4. Zinc finger binding sites also induce enhanced expression (Hossain et al., 2015). We introduced these *cis*-acting elements into the hybrid promoter FM′M. First, we placed four copies of the UAS at the 5′ end of the hybrid promoter; thus, in our constructs, UAS×4 functioned as a distal *cis*-acting element. The GAL4 binding domain UAS×4 has been used previously (Halpern et al., 2008; Zhu et al., 2020). To construct artificial transcription factors, we combined GAL4 with either VP16, a well-known activation domain from herpes simplex virus (HSV), or an activation domain of TAC3 (Sevin-Pujol et al., 2017). Expressing either of the two artificial transcription factors, GAL4:VP16 and GAL4:TAC3d2, with constructs driven by FM′M-UD influenced the levels of transcriptional activation, with GAL4:TAC3d2 producing a far greater effect than GAL4:VP16. This difference may result from the types of AD in these transcription factors. There are multiple types of AD, including acidic, glutamine-rich, proline-rich, and isoleucine-rich domains (Courey et al., 1989; Mermod et al., 1989; Attardi and Tjian, 1993). It has been shown previously that the activation domain of TAC3 activates transcription at both proximal and distal locations (Zhu et al., 2020); this is a convenient property when designing an artificial system for high level expression. Although both VP16 and TAC3d2 belong to the acidic domain group, they are known to differ in their effects. We confirmed that VP16 and TAC3d2 do indeed differ in their degree of activation.

We inserted the zinc finger binding site between the F and M′ fragments. The artificial transcription factor, ZinC7:TAC3d2, suppressed gene expression, even though the DBD ZinC7 contains the TAC3d2. The underlying cause of this suppression was not fully understood. ZinC7:TAC3d2 may have hindered the assembly of the transcriptional machinery. The binding affinity of ZinC7 to two copies of the zinc finger binding motif is very high (Kd = 0.46 nM) (Wu et al., 1995; Liu et al., 1997). Such tight binding of ZinC7/TAC3d2 may inhibit elongation of transcription by preventing the transcription machinery from moving along the promoter.

The role of the terminator is to generate the 3′ end at the correct position, which in turn leads to the proper addition of poly(A) to the 3′ end of the transcript; this ensures the stability of mRNA (Mandel and Tong, 2007; Kumar et al., 2019). Thus, the terminator also contributes greatly to gene expression levels (Nagaya et al., 2010). Many strong terminators have been identified, including those of Arabidopsis *HSP18* and *N. benthamiana extensin* (Nagaya et al., 2010; Rosenthal et al., 2018; De Felippes et al., 2022). Another approach is to use multiple terminators (Ingelbrecht et al., 1989; Nagaya et al., 2010). We explored the use of multiple terminators, together with a matrix attachment region (MAR). MARs at the 5′ or 3′ regions of a gene contribute to expression of that gene (Gasser et al., 1990; Boulikas, 1993). Often, a single MAR at either the 3′ or 5′ region of a gene is sufficient to induce high expression (Arope et al., 2013). RB7, a MAR from tobacco, contributes to high level expression of nearby genes (Halweg et al., 2005). The PINII terminator is a strong terminator (An et al., 1989), and the 35S terminator is used widely in expression vectors. We confirmed that combining these two terminators together with RB7 led to enhance expression compared to the terminator of *RD29B*, a gene that is highly induced following treatment with abscisic acid (Nakashima et al., 2006).

In conclusion, we designed a high expression system by combining a strong promoter and a strong terminator with a matrix attachment sequence and an artificial transcription factor. The robust promoter was generated by fusing together promoter fragments from three different viral promoters and adding heterologous *cis*-acting elements. The strong terminator was generated by fusing two terminators from PINII and 35S together with a matrix attachment sequence. An artificial transcription factor was produced using a strong plant activation domain from TAC3 and the DBD for yeast GAL4. When all these components were combined in a novel expression system, the production of recombinant hIL6 was 8-fold higher at protein level than that of CaMV 35S, yielding 43 mg/kg of recombinant protein.

## Data availability statement

The original contributions presented in the study are included in the article/**Supplementary Material**. Further inquiries can be directed to the corresponding author.

## Author contributions

IH and AY conceived conception of the study and wrote the manuscript. AY made novel hybrid promoter and artificial transcription factor and conducted experiments and analyzed results. JK made composite terminator. JL contributed to the creation of experimental concepts and the analysis of experimental results. S-JS contributed to vector construction. All authors contributed to the article and approved the submitted version.
