# Supplementary material for: Design of an artificial transcriptional system for production of high levels of recombinant proteins in tobacco (Nicotiana benthamiana)
Source: Front Plant Sci. 2023 Feb 23;14:1138089. doi: 10.3389/fpls.2023.1138089 (PMC9995837; doi:10.3389/fpls.2023.1138089)
Supplement: Supplementary file 1 [file DataSheet_1.docx]

**Supplemental figures**

**
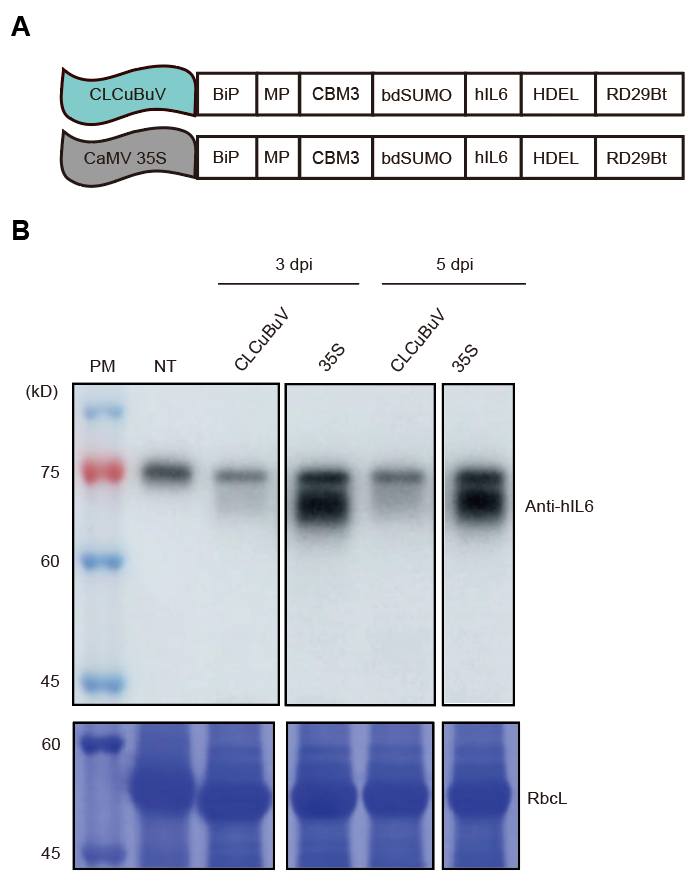
**

**Supplemental Figure 1. The CLCuBuV promoter from Cotton Leaf Curl Burewala Virus is not stronger than the CaMV 35S promoter in the production of recombinant proteins in *N. benthamiana*.**

(A) Schematic presentation of 2 expression vectors for a recombinant construct *BMCS:hIL6*. (B) Expression levels of *BMCS:hIL6*. The two expression vectors were introduced into leaf cells of *N. benthamiana* via *Agrobacterium*-mediated infiltration. Leaf tissues were harvested at 3 dpi and 5 dpi. Total soluble proteins from leaf tissues were analyzed by western blotting using anti-hIL6 antibody. An identical membrane was stained with CBB; RbcL was used as a loading control. PM, protein size standard; NT, wild-type plant without infiltration; 35S, CaMV 35S promoter.


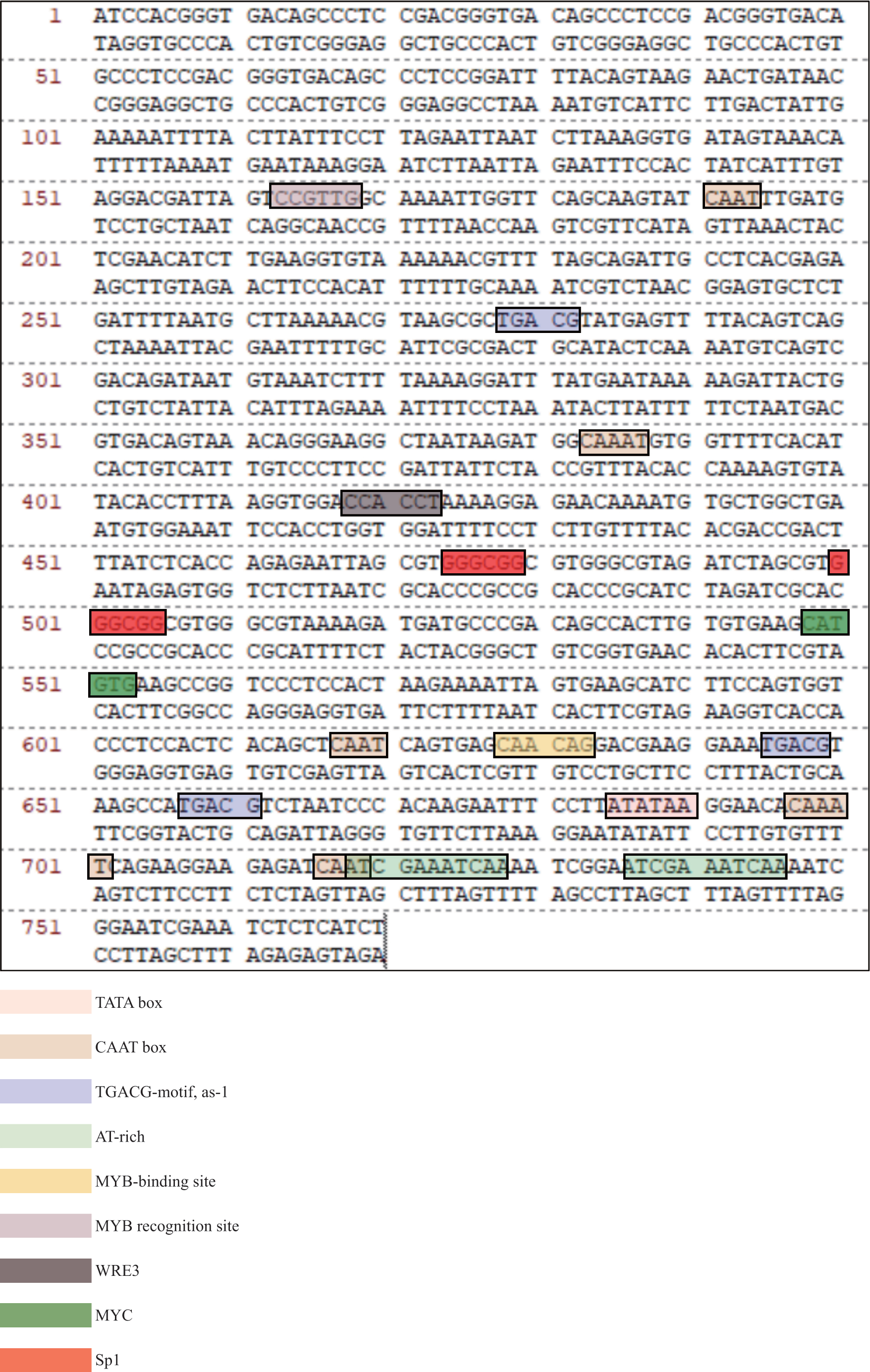


**Supplemental Figure 2. *In silico* analysis of *cis*-acting elements in the FMʹM-UD promoter**

*cis*-acting elements in the FMʹM-UD promoter identified using PlantCARE software. The colored sequences indicate the type and location of potential *cis*-acting elements.

**Supplemental Figure 3. SDS/PAGE analysis of unbound (UB), first wash-off (W1), and second wash-off (W2) fractions of MCC bead-based purification of hIL6 recombinant protein produced by the expression vectors CR, FR, and FR plus GAL4/TAC3d2**

(**A**) Schematic presentation of constructs used for expression of *BMCS:hIL6* recombinant protein. CR: CaMV 35S promoter::BMCS:hIL6 with RD29Bt; FR: FMʹM-UD promoter::BMCS:hIL6 with RD29Bt. GAL4:TAC3d2 is a transcription factor. (**B**) SDS/PAGE gel stained with CBB. PM: protein size standard; UB: unbound fraction; W1: the first wash-off fraction; W2: the second wash-off fraction.

**Supplemental Figure 4. SDS/PAGE analysis of unbound (UB), first wash-off (W1), and second wash-off (W2) fractions of MCC bead-based purification of hIL6 recombinant protein produced by the expression vectors CR, F3, and F3 plus GAL4/TAC3d2**

(**A**) Schematic presentation of constructs used for expression of *BMCS:hIL6* recombinant protein. CR: CaMV 35S promoter::BMCS:hIL6 with RD29Bt; F3: FMʹM-UD promoter::BMCS:hIL6 with 3PRt. GAL4:TAC3d2 is a transcription factor used with F3. (**B**) SDS/PAGE gel stained with CBB. PM: protein size standard; UB: unbound fraction; W1: first wash-off fraction; W2: second wash-off fraction.

**Supplemental Table 1. Sequences of the promoter and terminators.**

| **Promoter or terminator** | **Sequence** |
| --- | --- |
| MMV-FLt (-193 to +63) promoter | AAAAGATGATGCCCGACAGCCACTTGTGTGAAGCATGTGAAGCCGGTCCCTCCACTAAGAAAATTAGTGAAGCATCTTCCAGTGGTCCCTCCACTCACAGCTCAATCAGTGAGCAACAGGACGAAGGAAATGACGTAAGCCATGACGTCTAATCCCACAAGAATTTCCTTATATAAGGAACACAAATCAGAAGGAAGAGATCAATCGAAATCAAAATCGGAATCGAAATCAAAATCGGAATCGAAATCTCTCATCT |
| MMV-Sgt (-306 to -125) promoter | GTTTTACAGTCAGGACAGATAATGTAAATCTTTTAAAAGGATTTATGAATAAAAAGATTACTGGTGACAGTAAACAGGGAAGGCTAATAAGATGGCAAATGTGGTTTTCACATTACACCTTTAAGGTGGACCACCTAAAAGGAGAACAAAATGTGCTGGCTGATTATCTCACCAGAGAATT |
| FMV-Sgt (-270 to -63) promoter | TTTACAGTAAGAACTGATAACAAAAATTTTACTTATTTCCTTAGAATTAATCTTAAAGGTGATAGTAAACAAGGACGATTAGTCCGTTGGCAAAATTGGTTCAGCAAGTATCAATTTGATGTCGAACATCTTGAAGGTGTAAAAAACGTTTTAGCAGATTGCCTCACGAGAGATTTTAATGCTTAAAAACGTAAGCGCTGACGTATGA |
| CaMV 35S promoter | AGATTAGCCTTTTCAATTTCAGAAAGAATGCTAACCCACAGATGGTTAGAGAGGCTTACGCAGCAGGTCTCATCAAGACGATCTACCCGAGCAATAATCTCCAGGAAATCAAATACCTTCCCAAGAAGGTTAAAGATGCAGTCAAAAGATTCAGGACTAACTGCATCAAGAACACAGAGAAAGATATATTTCTCAAGATCAGAAGTACTATTCCAGTATGGACGATTCAAGGCTTGCTTCACAAACCAAGGCAAGTAATAGAGATTGGAGTCTCTAAAAAGGTAGTTCCCACTGAATCAAAGGCCATGGAGTCAAAGATTCAAATAGAGGACCTAACAGAACTCGCCGTAAAGACTGGCGAACAGTTCATACAGAGTCTCTTACGACTCAATGACAAGAAGAAAATCTTCGTCAACATGGTGGAGCACGACACACTTGTCTACTCCAAAAATATCAAAGATACAGTCTCAGAAGACCAAAGGGCAATTGAGACTTTTCAACAAAGGGTAATATCCGGAAACCTCCTCGGATTCCATTGCCCAGCTATCTGTCACTTTATTGTGAAGATAGTGGAAAAGGAAGGTGGCTCCTACAAATGCCATCATTGCGATAAAGGAAAGGCCATCGTTGAAGATGCCTCTGCCGACAGTGGTCCCAAAGATGGACCCCCACCCACGAGGAGCATCGTGGAAAAAGAAGACGTTCCAACCACGTCTTCAAAGCAAGTGGATTGATGTGATATCTCCACTGACGTAAGGGATGACGCACAATCCCACTATCCTTCGCAAGACCCTTCCTCTATATAAGGAAGTTCATTTCATTTGGAGAGAACACG |
| 3PR terminator | GTCCGCAAAAATCACCAGTCTCTCTCTACAAATCTATCTCTCTCTATTTTTCTCCAGAATAATGTGTGAGTAGTTCCCAGATAAGGGAATTAGGGTTCTTATAGGGTTTCGCTCATGTGTTGAGCATATAAGAAACCCTTAGTATGTATTTGTATTTGTAAAATACTTCTATCAATAAAATTTCTAATTCCTAAAACCAAAATCCAGTGACGAGCTCCTAGAGTCACCCTGCAATGTGACCCTAGACTTGTCCATCTTCTGGATTGGCCAACTTAATTAATGTATGAAATAAAAGGATGCACACATAGTGACATGCTAATCACTATAATGTGGGCATCAAAGTTGTGTGTTATGTGTAATTACTAATTATCTGAATAAGAGAAAGAGATCATCCATATTTCTTATCCTAAATGAATGTCACGTGTCTTTATAATTCTTTGATGAACCAGATGCATTTTATTAACCAATTCCATATACATATAAATATTAATCATATATAATTAATATCAATTGGGTTAGCAAAACAAATCTAGTCTAGGTGTGTTTTGCTAATTATTGGGGGATAGTGCAAAAAGAAATCTACGTTCTCAATAATTCAGATAGAAAACTTAATAAAGTGAGATAATTTACATAGATTGCTTTTATCCTTTGATATATGTGAAACCATGCATGATATAAGGAAAATAGATAGAGAAATAATTTTTTACATCGTTGAATATGTAAACAATTTAATTCAAGAAGCTAGGAATATAAATATTGAGGAGTTTATGATTGAGCTCACCAACTCGGTCCATTTGCACCCCTAATCATAATAGCTTTAATATTTCAAGATATTATTAAGTTAACGTTGTCAATATCCTGGAAATTTTGCAAAATGAATCAAGCCTATATGGCTGTAATATGAATTTAAAAGCAGCTCGATGTGGTGGTAATATGTAATTTACTTGATTCTAAAAAAATATCCCAAGTATTAATAATTTCTGCTAGGAAGAAGGTTAGCTACGATTTACAGCAAAGCCAGAATACAAAGAACCATAAAGTGATTGAAGCTCGAAATATACGAAGGAACAAATATTTTTAAAAAAATACGCAATGACTTGGAACAAAAGAAAGTGATATATTTTTTGTTCTTAAACAAGCATCCCCTCTAAAGAATGGCAGTTTTCCTTTGCATGTAACTATTATGCTCCCTTCGTTACAAAAATTTTGGACTACTATTGGGAACTTCTTCTGAAAATAGTG |

**Supplemental Table 2. The sequences of primers used in this study.**

| **Primer** | **Sequence** |
| --- | --- |
| MMSg-F | AACTGCAGGTTTTACAGTCAGGACAGATAATGTAAA |
| MMFg-F | AACTGCAGAAAAGATGATGCCCGACAGCCAC |
| FsMf-R | TCATACGTCAGCGCTTACGTTTTTAA |
| FsMf-F | TTAAAAACGTAAGCGCTGACGTATGAAAAAGATGATGCCCGACAGCC |
| MFM-R | AATTCTCTGGTGAGATAATCAGCC |
| MFM-F | GGCTGATTATCTCACCAGAGAATTTTTACAGTAAGAACTGATAACAAAAATTTTACTTATTTC |
| FMM-R | GCTCTAGAAGATGAGAGATTTCGATTCC |

***Primer sequence for motif insertion**

| **Primer** | **Sequence** |
| --- | --- |
| FMM-U-F | AACTGCAGATCCACGGGTGACAGCCCTCCGACGGGTGACAGCCCTCCGACGGGTGACAGCCCTCCGACGGGTGACAGCCCTCCGGATTTTACAGTAAGAACTGATAACAAAAATTTTA |
| FMM-US-F | AGCGTGGGCGGCGTGGGCGTAAAAGATGATGCCCGACAGCCACTTGTGTGAAGCAT |
| FMM-US-R | ACGCCCACGCCGCCCACGCTAATTCTCTGGTGAGATAATCAGCCAGCA |
| FMM-UD-F | AGCGTGGGCGGCGTGGGCGTAGATCTAGCGTGGGCGGCGTGGGCGTAAAAGATGATGCCCGA |
| FMM-UD-R | ACGCCCACGCCGCCCACGCTAGATCTACGCCCACGCCGCCCACGCTAATTCTCTGGTGAGATAATCAGCCA |

***Primer sequence for GOI and terminators**

| **Primer** | **Sequence** |
| --- | --- |
| XbaI-5`UTR-BIP-F | GGTCTAGAATTATTACATCAAAACAAAAAATGGCTCGCTCG |
| GFP-HDEL-XhoI-R | CCCTCGAGCTAGAGCTCATCGTGCTTGTACAG |
| 6*His-HDEL-XhoI-R | CCCTCGAGTTAAAGCTCATCATGGTGGTGATGGTG |
| hIL6-HDEL-XhoI-R | CCCTCGAGCTAGAGCTCATCGTGCATCTGCC |
| XhoI-RD29Bt-F | CCCTCGAGAATTTTACTCAAAATGTTTTGGTTGCTATG |
| EcoRI-RD29Bt-R | GGGAATTCATTTTTGTTTGAACTAATCAACATCTTAATTAATAATTCTAC |
| XhoI-3PRt-F | CCCTCGAGGTCCGCAAAAATCACCAGTC |
| 3PRt-EcoRI-R | GGGAATTCCACTATTTTCAGAAGAAGTTCCCAATAGT |

***Primer sequence for qRT-PCR**

| **Primer** | **Sequence** |
| --- | --- |
| hIL6-F | GGCTCTCGCAGAAAATAACCTTAA |
| hIL6-R | GAAACCAGACTGAAAACATCCATCT |
| HygR-F  HygR-R | GGTGTCGTCCATCACAGTTT  CCGCAAGGAATCGGTCAATA |
